# Phylogeography of HIV-1 suggests that Ugandan fishing communities are a sink for, not a source of, virus from general populations

**DOI:** 10.1038/s41598-018-37458-x

**Published:** 2019-01-31

**Authors:** Nicholas Bbosa, Deogratius Ssemwanga, Rebecca N. Nsubuga, Jesus F. Salazar-Gonzalez, Maria G. Salazar, Maria Nanyonjo, Monica Kuteesa, Janet Seeley, Noah Kiwanuka, Bernard S. Bagaya, Gonzalo Yebra, Andrew Leigh-Brown, Pontiano Kaleebu

**Affiliations:** 1 Medical Research Council/Uganda Virus Research Institute and London School of Hygiene & Tropical Medicine Uganda Research Unit, Entebbe, Uganda; 2 School of Public Health, College of Health Sciences, Makerere University, Kampala, Uganda; 3 Department of Immunology and Molecular Biology, School of Biomedical Sciences, College of Health sciences, Makerere University, Kampala, Uganda; 4 The Roslin Institute, University of Edinburgh, Edinburgh, UK; 5 Institute of Evolutionary Biology, University of Edinburgh, Edinburgh, UK

## Abstract

Although fishing communities (FCs) in Uganda are disproportionately affected by HIV-1 relative to the general population (GP), the transmission dynamics are not completely understood. We earlier found most HIV-1 transmissions to occur within FCs of Lake Victoria. Here, we test the hypothesis that HIV-1 transmission in FCs is isolated from networks in the GP. We used phylogeography to reconstruct the geospatial viral migration patterns in 8 FCs and 2 GP cohorts and a Bayesian phylogenetic inference in BEAST v1.8.4 to analyse the temporal dynamics of HIV-1 transmission. Subtype A1 (*pol* regio*n*) was most prevalent in the FCs (115, 45.1%) and GP (177, 50.4%). More recent HIV transmission pairs from FCs were found at a genetic distance (GD) <1.5% than in the GP (Fisher’s exact test, p = 0.001). The mean time depth for pairs was shorter in FCs (5 months) than in the GP (4 years). Phylogeographic analysis showed strong support for viral migration from the GP to FCs without evidence of substantial viral dissemination to the GP. This suggests that FCs are a sink for, not a source of, virus strains from the GP. Targeted interventions in FCs should be extended to include the neighbouring GP for effective epidemic control.

## Introduction

Human immunodeficiency virus type 1 (HIV-1) prevalence and incidence is higher among certain populations relative to other groups in Uganda. Among these, the fisher folk (FF) and female sex workers have the highest documented HIV-1 incidence rates^1^. An earlier report showed that majority of new HIV-1 infections in key populations were likely to come from the fishing communities (FCs)^2^ while a cross-country analysis^3^ among most-at-risk-populations in developing countries revealed that FF had the highest HIV-1 prevalence relative to other high-risk groups and the general population (GP).

“Fishing communities” is a general term used in this study to refer to groups of persons living in villages that are located along the shores of Lake Victoria or on islands and who are largely dependent on the harvest or processing of fishery resources to meet their social and economic needs^1^. In contrast, GP refers to people living mostly on the mainland or in towns adjacent to the FCs (approximately 10–40 kms) who do not derive their livelihood primarily from fishing-related activities^4^. HIV prevalence in the FCs is very high; estimated at about 29%^5^ and reaching as high as 40% in some communities^5^. These figures significantly exceed the national average of 7.3%^6^. Annual incidence rates of up to 6/100 person-years at risk (PYAR)^4^ have been reported among high-risk individuals in the FCs, which is much higher than the national estimated rate of 1/100 PYAR^4^. The high incidence rates have been attributed to risky sexual behaviour involving multiple partnerships, high alcohol consumption, low condom use, limited access to health services and transactional sex^7–9^.

The FF, in light of recent reports of high HIV-1 incidence rates have become an important population in planning informed prevention strategies^1^. This is largely due to the perceived potential for new HIV-1 infections to spread from the FCs to the GP and thus impeding preventative efforts centred on the general population^10^. However, the patterns of HIV-1 transmission in the FCs are not well enough understood to give high confidence that the implementation of any specific transmission interventions would be effective^11,12^.

Transmission network studies are vital in identifying traits associated with onward viral transmission among high-risk groups and understanding disease spread and control^13^ but are still scarce in sub-Saharan countries^10,14–17^. Current pilot studies by the MRC/UVRI and LSHTM Uganda Research unit are directed towards implementing combination prevention measures in the FCs yet for prevention to be effective^18^, the transmission dynamics need to be understood. We previously used phylogenetic techniques to identify transmission clusters in recently infected HIV-1 FF of Lake Victoria^16^ and to reconstruct the historical initial introduction and spread of HIV-1 in Uganda within our high-risk cohorts^19^. Our recent study applied phylogenetic and epidemiological approaches to identify factors contributing to the ongoing epidemic in the FCs of Lake Victoria and found a majority of transmission linkages (83%) to occur within communities^11^. However, the role of viral introductions from outside the FCs and temporal dynamics of HIV transmission in identified networks were not evaluated. In the present study, we set out to test the hypothesis that HIV transmission in the FCs is isolated from networks in the neighbouring GP. We used phylogeography to reconstruct the viral migration patterns between the two populations and a phylodynamic analysis in the BEAST program to determine the temporal dynamics of HIV transmission.

## Results

### HIV Subtyping

HIV-1 partial *pol* sequences (n = 606) from the FCs and GP were classified (Table 1). The population subgroups did not differ significantly in subtype prevalence as shown below.

**Table 1:** Distribution of HIV-1 *pol* Sequences According to Subtype and Cohort.

HIV Subtype	Database sequences (FCs)	FCs	GP	Total	P-values^‡^ (Proportions in FCs vs GP)
Subtype A1	15 (34.1%)	100 (47.4%)	177 (50.4%)	292	0.24
Subtype D	14 (31.8%)	70 (33.2%)	122 (34.8%)	206	0.35
Subtype C	1 (2.3%)	2 (0.9%)	9 (2.6%)	12	0.08
Inter-subtype recombinants	14 (31.8%)	39 (18.5%)	43 (12.2%)	96	0.02^*^
Total	**44**	**211**	**351**	**606**	

^‡^P-values according to the two-sample test of proportions.

^*^Significant difference in inter-subtype recombinants proportion; although the lower limit of the confidence interval (CI) is very close to zero, 95% CI (0.0004–0.126).

### Network analysis

Eighty-one cases were linked at a maximum pairwise GD of 4.5% (>0.95 bootstrap support) as 35 pairs, 2 triplets and 1 cluster of 5 individuals (Supplementary Table S1). At a more stringent GD cut-off of 1.5% (>0.95 bootstrap support), 13 pairs were identified (Supplementary Table S2) of which 10 (76.9%) were from the FCs and 3 (23.1%) from the GP. At a GD cut-off of 4.5%, an additional 18 linked pairs belonging to pure viral subtypes were found. Of these, 15 were in the GP and only 3 in the FCs. In Table 2 below, there were more pairs in the FCs (n = 10) at a GD of <1.5% than in the GP (n = 3) but fewer (3 in FCs and 15 in GP) above a 1.5% GD threshold showing that while older transmission networks can be detected in the GP, any linkage found among individuals in the FCs is more likely to be recent (Table 2, Fisher’s exact test p = 0.001).

**Table 2:** Contingency table showing pure subtype pairs identified at GD thresholds of 1.5%-4.5% and <1.5% according to population subgroups.

Group	Number of pairs 1.5–4.5%	Number of pairs <1.5%	Total
FCs	3	10	13
GP	15	3	18
Total	**18**	**13**	**31**

Fisher’s Exact Test p = 0.001.

As a sensitivity analysis, we also analysed the data using HIV-TRACE. HIV-TRACE and Cluster Picker (CP) results were similar for pairs identified, based on GD (cut-off = 1.5%). We observed 27 pairs (21 FCs, 6 GP) using HIV-TRACE in comparison to 24 pairs (19 FCs, 5 GP) using CP. Overall, HIV-TRACE detected 27 pairs (21, 77.8% from FCs and 6, 22.2% from the GP) and 2 clusters (1 cluster of triplets and another cluster of 4 individuals). The 2 clusters identified were both from the FCs. In CP, 24 pairs (19 from FCs, 79.2% and 5, 20.8% from the GP) and 1 cluster of triplets (FCs) were identified. As expected, network analysis at a GD cut-off of 4.5% identified larger clusters and fewer pairs at this higher GD threshold in HIV-TRACE^20^; these included 2 very large clusters (1 cluster of 246 linked individuals and another cluster of 192 individuals), a cluster of triplets and 11 pairs (data not shown). Differences in results obtained using HIV-TRACE and CP arise from the use by HIV-TRACE of a single-linkage approach^21^ while the CP groups on the basis of maximum GD and requires a pre-specified bootstrap support.

### Time to Most Recent Common Ancestor (TMRCA) across occupation groups

The TMRCA for all pairs and clusters was estimated from the BEAST trees. Networks of individuals involved in fishing-related activities, farmers and bar attendants and women engaged in sex work (average age = 35 years) had average TMRCA of 3.1 (95% CI 0.3–5.9), 7.3 (95% CI 4.1–10.5), 6.1 (95% CI −8.9–21) and 10.6 (95% CI 2.8–18.5) years respectively. No significant difference (ANOVA; p = 0.1087) was observed in TMRCA between occupation groups although individuals involved in fishing-related activities were associated with shorter TMRCAs.

### Estimated viral transmission times

The time depth (TD) in years for clusters/pairs (Supplementary Table S3) provided an approximation to the time of transmission, in that it gives the time to the last common ancestor of the viral strains in the transmitter. The TD for 11 pairs (GD cut-off = 1.5%) (2 A1/D recombinant pairs excluded from the Bayesian phylodynamic analysis) was 2 years on average (range: 0.3–8.4) with 6 pairs from the FCs having a TD of ≤ 1 year (average = 0.53 years, range: 0.3–1) and 5 pairs from the GP with a TD of ≥ 1 year (average = 4.1 years, range: 1–8) (unpaired t-test; p = 0.0076, 95% CI 1.208–5.926).

### Phylogeographic analysis

**Figure 1:**
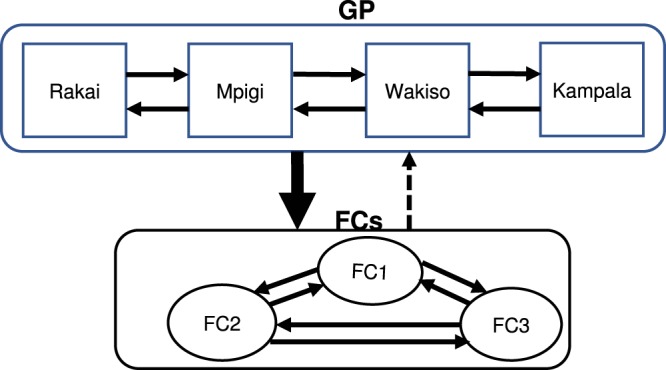
Schematic diagram showing statistically significant viral dissemination within and between the GP and FCs. The arrows show the direction of viral migration with the thicker arrow representing stronger support for transitions (BF > 10) between the 2 populations and the dotted arrow indicating non-significant (BF < 3) viral migration.

Strong support (Bayes Factor (BF) >10) for viral migrations inferred from BEAST location-annotated MCC trees (Supplementary Fig. S1) was observed between Rakai and Kampala along the Kampala-Masaka highway. Other significant transitions within FCs and the GP (BF > 3) are shown in Supplementary Table S4. A second phylogeographic analysis that excluded the background sequences was performed to determine whether they introduced a bias to the observed viral diffusion pattern. Results from this analysis showed very strong support for viral migrations (BF > 50) from the neighbouring GP to the FCs (FC1, FC2 and FC3) (Supplementary Table S5). In interpreting BF test results, a particular rate was considered significant if BF > 3 and strong if BF > 10^22^. Figure 1 below shows a summary of the viral migration patterns from the phylogeographic analysis.

## Discussion

In this study, we analyzed nucleotide sequences from the FCs and the neighbouring GP to reconstruct the geospatial and temporal dynamics of HIV-1 in transmission networks using a phylogeographic and phylodynamic approach. Of all sequences classified from both the FCs and GP, based on the HIV *pol* region, HIV-1 subtype A1 was the predominant subtype (49%) followed by subtype D (34%), inter-subtype recombinants (15%) and subtype C (2%). We note that earlier studies^16,19^ conducted in Lake Victoria FCs found subtype D to be the most common subtype. HIV-1 subtype A1 was the dominant subtype in identified networks (Supplementary Tables S1 and S2) with more recent viral transmission compared to subtype D. This is consistent with findings from a recent study that found subtype A as the predominant subtype (58%) among high-risk FCs followed by subtype D (39%) with less likely clustering of subtype D compared to subtype A^11^. A general increase in HIV-1 subtype A1 prevalence has been reported in the nearby area of Rakai^23^ and attributed to an apparently lower transmissibility of subtype D compared to subtype A1. This could indicate changing dynamics in the distribution of subtype diversity with implications for future vaccine development however, this increase has not been observed in studies within our cohorts^24,25^.

Our results highlight the role of recent HIV-1 infections in transmission networks among a largely heterosexual adult population (average age 35 years) involved in fishing-related activities, farming, bar work and commercial or transactional sex. Identifying networks at a more conservative GD cut-off of 1.5%^11,26^ allowed the detection of sequence pairs that represent recent HIV transmission in these populations^11^. The viral divergence times estimated from the TMRCA and TD revealed recent ongoing transmission in at least half of the pairs, mostly those from the FCs. This is in agreement with findings from our recent study that found at least 32% of identified transmission clusters in the FCs to be potentially recently infected with 36% of these characterized as incident-incident viral transmissions^11^. HIV-1 sequences from the FCs were thus associated with shorter TMRCAs and relatively low pairwise genetic distances.

Phylogeographic analysis showed strong support for viral migration (BF > 50) from the neighbouring GP to the FCs. Moreover, relaxing the GD threshold to 4.5% added relatively few additional pairs or clusters in the FCs, indicating a relatively unstable population with low residency. In a study that assessed the association between HIV-1 incidence and migration in a rural population in Rakai district, high HIV-1 incidence was found among recent migrants within the first 2 years^27^. Mobility has been reported to be an important driver for HIV transmission^28,29^. In respect of the results presented here, this implies that high levels of movement from the GP to the FCs as well as among FCs could be associated with the high incidence there. Furthermore, strong support for viral migration was found between Rakai and Kampala along the Kampala-Masaka highway. The Kampala-Masaka highway connects to the trans-African highway that was believed to have played a key role in the early spread of the HIV-1 epidemic and extends beyond Rakai district in South West Uganda. This area was associated with the first documented HIV AIDS case reported in a fishing village^30^ and is the historical epicenter of the HIV-1 epidemic in Uganda^19^. The Kampala-Masaka highway also provides an active transport network linking FCs in rural Mpigi and urban Wakiso district to Kampala, with several hotspots along this transport corridor such as Lukaya that are popular areas for long-distance truckers, female sex workers, road side bars and lodges. This could explain the strongly supported viral migration along this route. While previous studies^14,15,19^ have found minimal inter-population mixing between FCs and other communities however in this study we observed a significant level of viral diffusion between the adjacent GP and FCs that is most likely facilitated by these major highways.

Study limitations included a lower number of HIV sequences obtained from some of the study sites and genotyping was restricted to the HIV-1 *pol* fragment generally used for clinical screening of drug resistance mutations.

## Materials and Methods

### Ethical statement

This study was approved by the Uganda Virus Research Institute Research and Ethics Committee (GC/127/14/09/428) and by the Uganda National Council for Science and Technology (HS 1432). All procedures were performed in accordance with approved guidelines and regulation. All subjects provided written informed consent before they participated in the study.

### Study design

A cross-sectional study was carried out in 8 FCs and 2 GP (rural/urban) cohorts. Study participants were enrolled between September 2014 and September 2016 and completed structured questionnaires that captured general demographic, socioeconomic, partnership histories and behavioural data. The study inclusion criteria involved recruitment of HIV-1 positive individuals above 18 years of age. A biometric fingerprint-scanning device was used on all study participants to avoid duplicate enrolments.

### Study population and sample collection

A total of 606 HIV-1 partial *pol* sequences (mean length 1,257 bp) were analysed by phylogenetic methods. The sequences were part of the HIV-1 Molecular Epidemiology study that aimed to determine HIV-1 subtypes and transmission linkages among both high-risk and general populations in Uganda. Sequences from the FCs (n = 255) were of individuals from the HIV Combination Intervention (HIVCOMB) (n = 211) cohort and a cohort of recently infected FF (n = 44). In the HIVCOMB FF cohort, serial cross-sectional surveys were carried out in 3 FCs where combination intervention was implemented in intervention areas and deferred in the control areas for a period of 18 months but continued after completion of the study. Our second FF cohort^16^ consisted of initially uninfected HIV seronegative individuals (n = 1,000) followed up for a period of 18 months and samples collected from recent seroconverters at 6 monthly visits from 5 FCs in central and south western Uganda. Sequences (n = 351) from the GP comprised of HIV positive individuals who received care at health facilities adjacent the FCs but included patients diagnosed during the voluntary counselling and testing (VCTs). A map of the study sites is not shown because most of the FF lived in relatively small fishing villages where individuals could be identified. The names of the FCs were anonymized in this study to avoid breaching study participant confidentiality.

The number of HIV sequences contributed per site included 70 from each of 2 FCs, 71 from 1 FC and an additional 44 sequences from 5 communities of recently infected FF. Two GP sites had 200 and 151 sequences. Some FCs were located approximately 25–40 kms from the Kampala-Masaka highway while others were located approximately 5–12 kms from the Kampala-Entebbe highway. These included 7 sites on the mainland shores between Masaka and Entebbe and 1 site on an island 20 km from the northern shore of Lake Victoria. The FCs are located in Mpigi, a rural district (1,208 km^2^) located in central Uganda with a population of 251,512^31^, Wakiso, an urban district (1,907 km^2^) bordering Kampala in the northeast with a population of about 2 million^31^, in Masaka district (1,296 km^2^) located southwest of Kampala with a population of 296,649^31^ and Kalungu, a rural district (812 km^2^) bordered by Mpigi district to the east and Masaka district to the south with a population of 184,131^31^.

### HIV sequencing

Partial sequences of the HIV-1 *pol* gene as used for drug resistance testing were obtained. Such sequences are extensively used in transmission network studies^32^. This is because HIV-1 *pol* sequence fragments have been shown to accurately reconstruct viral phylogenies for the inference of HIV transmission dynamics^32,33^. Proviral DNA extracted from cell pellets using the QIAamp Viral DNA kit (Qiagen, Hilden, Germany) was used as PCR starting material to increase the amplification and sequencing success rate in samples from patients with a low-level viremia, as may apply if the individuals are receiving antiretroviral therapy. Nested PCR was performed to amplify the HIV-1 *pol* (protease codon 1–99 and the amino terminus of reverse transcriptase codons 1–320) using gene specific primers as described elsewhere^16^. Genotyping of the amplified products was done by sequencing of the purified fragment using the Big Dye Terminator v3.1 Cycle Sequencing Kit (Applied Biosystems) and results were analyzed using the ABI 3130 Genetic Analyzer (Applied Biosystems, Foster City, CA) as previously described^16^. Raw sequence data was edited using sequencher v4.10.1 (Gene codes Corporation, Ann Arbor, MI).

HIV-1 nucleotide sequences were analysed to classify HIV variants circulating in the two populations and determine the predominant strains to be included in the phylogeographic analysis, identify genetically linked sequences as per a set GD threshold, implement a Bayesian phylogenetic approach to determine the time related to HIV transmission of individuals in networks (temporal dynamics) and to infer the direction of viral transmission between the FCs and GP (spatial dynamics). In order to examine the spatial dynamics, we included information on the geographic areas from where the sequences were obtained as described in details below.

### HIV subtyping

HIV sequences were classified using COMET^34^ and SCUEAL^35^ programs. Subtyping results that were discordant in COMET and SCUEAL were analysed in REGA v3^36^.

### Transmission network analysis

Duplicate sequences (n = 2) were removed using the ElimDupes tool^37^ to ensure that only 1 sequence per individual was included in the dataset. Multiple sequence alignments were done using MUSCLE^38^ and edited in Geneious v9.0.5^39^. A maximum likelihood (ML) phylogenetic tree was constructed using the randomized Accelerated Maximum Likelihood (RAxML) program^40^ with a general time reversible (GTR) model of nucleotide substitution that is Gamma distributed and determined as the fittest model by the Akaike Information Criteria (AIC) in Jmodeltest^41^. Transmission networks were identified on the ML tree using Cluster Picker (CP) v1.2.2^42^, initially at a maximum pairwise GD of 4.5% (>95% bootstrap support) and then at a GD cut-off of 1.5%^26^. A separate cluster detection program, HIV-TRACE^43^ that calculates Tamura-Nei (TN 93)^44^ pairwise genetic distances between sequences and employs a single-linkage algorithm to detect transmission chains was used along with Cluster Picker to minimize bias in cluster detection and as a sensitivity analysis^45^. The stepwise approach of first identifying larger long-lasting phylogenetic clusters at higher GD thresholds followed by the detection of active transmission chains at a lower GD cut-off has been suggested in literature^46^. The upper GD limit for detecting HIV transmission clusters using *pol* sequences has been estimated at around 4.5%^42^. Above this threshold, the number and size of clusters detected stays almost constant^42^ although below this cut-off, establishing a GD threshold varies according to the study goals^20^. The goal of using a 1.5% GD cut-off was to identify pairs associated with more recent HIV infection which has been associated with higher mobility^27^ that could have an impact on directional transmission of HIV between populations. A GD threshold of 1.5% was the preferred threshold for the identification of transmission networks associated with recent HIV-1 infection in this population^11^. Phylogenetic transmission networks were defined as genetically closely related HIV-1 sequences based on a GD threshold that formed monophyletic groups on the phylogenetic tree with high support (>0.95) where 2 highly similar sequences were referred to as pairs and >2 sequences as clusters. Results were viewed in FigTree v1.4.2^47^. Participant records were anonymized by assigning new unique identifiers which were used for all analyses to prevent identification of individuals in transmission networks.

### Bayesian phylogenetic inference to estimate HIV-1 transmission times

Sequences classified as pure A1 and D subtypes were analyzed in BEAST^48^. BEAST is a Bayesian statistical inference that incorporates a wide range of evolutionary, demographic and nucleotide substitution models for hypothesis testing and inferring evolutionary dynamics of samples in a population being investigated. A Bayesian Markov Chain Monte Carlo (MCMC) method was implemented in BEAST v1.8.4 for 300 million generations sampling after every 10,000th iteration. We used an uncorrelated lognormally-distributed relaxed molecular clock coupled with the SRD06 model of nucleotide substitution^19,22,49^ and a coalescent skygrid tree prior^50–52^. Marginal likelihood estimates of different substitution models that included the SRD06^49^ and Yang 96^53^, demographic models (Bayesian Skygrid and GMRF Skyride) and molecular clocks (strict and relaxed) were compared using the path sampling/stepping-stone method^54^ to determine the models that best fitted the data. A lognormal prior distribution was specified for the evolutionary rate mean (ucld.mean; initial value = 1, mean = 0 and stdev = 1.0) and a normal prior distribution for the evolutionary rate standard deviation (ucld.stdev; initial value = 0.3, mean = 0.3 and stdev = 1.0). An evolutionary rate of 1.5 × 10^−3^ substitutions/site/year was expected based on estimates from a previous study^19^. Convergence of the MCMC results was examined in TRACER^55^ based on the effective sample size (ESS) of parameter estimates after a 20% burn-in. Maximum Clade Credibility (MCC) trees were generated with TreeAnnotator^56^ and visualized in FigTree. To approximate the time to HIV transmission between linked individuals in networks, the time to the most recent common ancestor (TMRCA) for each cluster/pair was first determined. This was computed as the difference between the date in calendar years of the most recent terminal node or tip on the MCC tree and the node height. A TD or node age was then determined as the difference between the TMRCA at the common node and the most recent sample date within a pair or cluster.

### Phylogeographic analysis

This analysis was based on the reconstruction of ancestral states and the count of the number of location changes that occurred in phylogenies. Ancestral state reconstruction (ASR) generally refers to the process of annotating the internal nodes of the tree with inferred information about the unsampled organisms they represent and aims to assign the character states of the ancestor organisms. A Parsimony algorithm that minimizes the number of character state changes on a phylogenetic tree has the advantage of being fast and simple to implement^57,58^. However, this method is dependent upon the accuracy of a single tree and therefore requires an explicit model of evolution for optimum results. In contrast, the ASR used in this study (in BEAST) accounts for uncertainty in tree reconstruction by allowing for character state changes to be inferred over a set of several posterior trees. It is based on a Markov model that describes a probabilistic process of proposing a new state, calculating its acceptance probability and accepting or rejecting the proposed state in a repetitive sequence^59^. A variety of models that include diffusion^60^ and structured coalescent models^61,62^ were used to merge lineages backwards in time to the most recent common ancestors at the internal nodes and attain a description of the viral migration process between locations.

Partial HIV-1 pol sequences from Uganda belonging to A1 (n = 170) and D (n = 230) subtypes were downloaded from the Los Alamos National Laboratory (LANL) HIV sequence database with sampling dates (1992–2006) and location information (mostly Kampala, Wakiso and Rakai). Additional background sequences from the LANL database were included to avoid inferring false links during the phylogeographic analysis, a common anomaly in phylogenetic-based analyses. Adding historical samples to phylogeographic analyses has been shown to improve ASR and convergence of the MCMC chains^19^. Further analysis on all sequences was done in ViroBLAST^63^ to retrieve only those that were similar to the query sequences (≥95% bootstrap support) as previously described^11^. The datasets were analyzed in TempEst v1.5^64^ to exclude sequences (n = 4) with high evolutionary rates whose genetic divergence was incongruent with their sampling times.

A phylogeographic analysis was performed in the BEAST program that included 7 locations namely: 3 FCs (FC1, FC2 and FC3), 2 GP sites (Mpigi and Wakiso) and background sequences from other locations (Kampala and Rakai) (Supplementary Table S6). Phylogeography generally describes the geographical distribution of lineages and has been used to reconstruct the geospatial dynamics of disease spread or viral migration while simultaneously allowing for temporal information to be obtained from time-measured phylogenies^60^. By considering geographic locations as discrete states in a Bayesian statistical framework, we are able to infer the evolutionary history of viral migration through time and colour the tree branches by location both at the tips where it is known and at the internal nodes where it is inferred using an ASR. We used an asymmetric substitution model and a strict molecular clock and applied the Bayesian Stochastic Search Variable selection (BSSVS) method to identify the number of non-zero transitions (migrations) rates between states and generate a Bayes factor (BF) test^60^. A BF test was used to assign statistical support for location changes that occurred more frequently on the trees and to determine the most parsimonious depiction of the viral migration patterns^64^. The direction of transition between the states (locations) was inferred using the asymmetrical discrete traits analysis implemented in the BEAST program^19^. To prevent the potential bias caused by over-sampling a particular location, sequences from each location were subsampled and locations with minimal sampling coverage (<10 sequences) were excluded from the analysis. Phylogeographic analysis is sensitive to sampling whereby a very small sample size might not yield sufficient information to describe the inferred migration profiles while a very large sample size would overwhelm the transition matrix. It is therefore essential that the sampling strategy ensures a sufficiently representative and proportional number of samples from each of the locations to avoid over scoring transitions or counts in the tree. As a result, over-sampled locations in comparison to other sites might require down sampling to avoid bias while those that are under-represented might be of little benefit and could be excluded from the analysis^65^. The viral migration patterns were reconstructed in the SPREAD program^66^. SPREAD is an acronym for Spatial Reconstruction of Evolutionary dynamics, a program that was developed to aid in the analysis and visualization of Bayesian phylogeographic reconstructions such as those generated from BEAST. A migration matrix with non-zero values for significant migrations between locations is generated with a BF test. These two programs enable phylogeographic inferences to be done in natural time scales.

### Statistical analysis

STATA version 13 (College Station, TX: StataCorp LP) was used to compare proportions of subtype prevalence in the FCs and GP using a two-sample test of proportions. The Fisher’s exact test was used to compare the number of pure viral subtype transmission pairs at different GD thresholds in the FCs and GP. P-values < 0.05 were considered to be statistically significant.

### Accession codes

Genbank accession numbers: MG434786-MG435347. For database sequences: JX498971–JX498972, JX498976-JX498990 and JX498992-JX499018.

## Supplementary information

Supplementary Information

## Data Availability

The datasets generated during and/or analysed during the current study are available from the corresponding author on reasonable request.
