# Supplementary material for: Phylogeography of HIV-1 suggests that Ugandan fishing communities are a sink for, not a source of, virus from general populations
Source: Sci Rep. 2019 Jan 31;9:1051. doi: 10.1038/s41598-018-37458-x (PMC6355892; doi:10.1038/s41598-018-37458-x)
Supplement: Supplementary file 1 — Supplementary Information [file 41598_2018_37458_MOESM1_ESM.pdf]

Phylogeography of HIV-1 suggests that Ugandan fishing communities are a sink for, not a source of, virus from general populations

Nicholas Bbosa, Deogratius Ssemwanga, Rebecca N. Nsubuga, Jesus F. Salazar-Gonzalez, Maria Salazar, Maria Nanyonjo, Monica Kuteesa, Janet Seeley, Noah Kiwanuka, Bernard S. Bagaya, Gonzalo Yebra, Andrew Leigh-Brown and Pontiano Kaleebu.

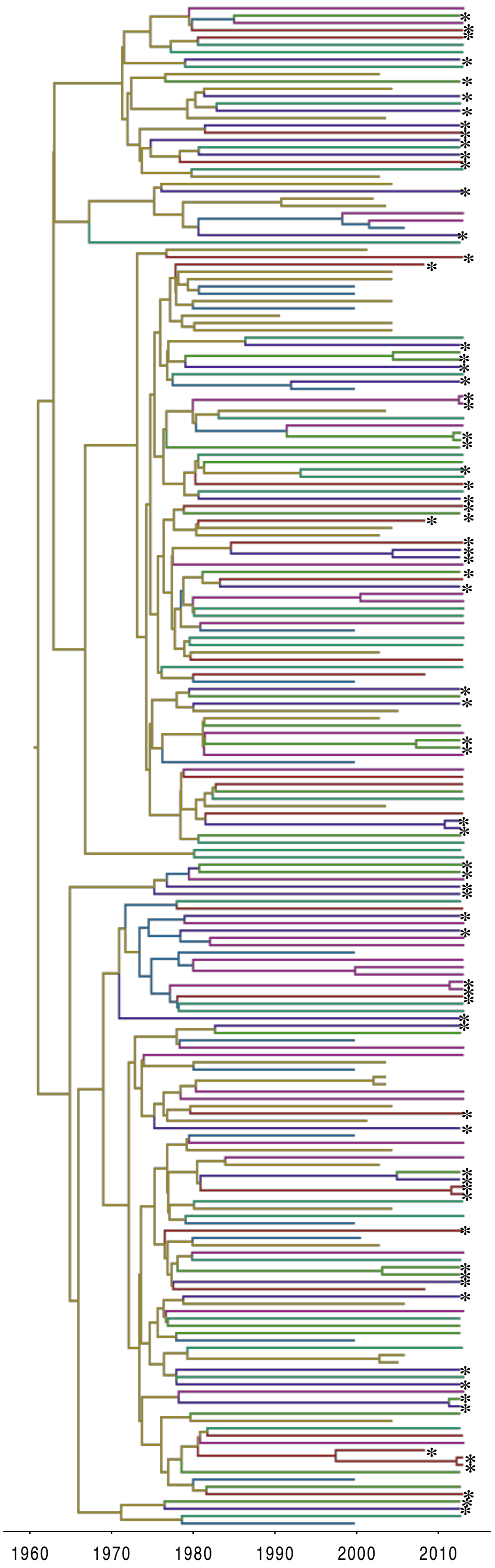

Figure S1: Simplified location-annotated Maximum Clade Credibility (MCC) tree. The branches on the tree are colored according to location with the FCs indicated by asterisks (*) at the branch tips. Time scale at the bottom is in calendar years.

Table S1. Summary of HIV-1 transmission network characteristics at a maximum pairwise genetic distance of 4.5%

**HIV-1 variant**	**Cluster Size**			**FCs**		**GP**		**Linked pairs/clusters by gender**				
	2	3	5	Pairs	Clusters	Pairs	Clusters	M-F	F-F	M-M	M-F-F	M-M-F-F-F
Subtype A1	19	1	_	9	1	10	_	12	4	3	1	_
Subtype D	11		_	4	_	7	_	7	4	_	_	_
Subtype C	1	_	_	_	_	1	_	1	_	_	_	_
A1/D recombinants	4	_	1	4	1	_	_	4	_	_	_	1
Other inter-subtype recombinants	_	1					1				1	
**Total**	**35**	**2**	**1**	**17**	**2**	**18**	**1**	**24**	**8**	**3**	**2**	**1**

Table S2. Summary of HIV-1 transmission network characteristics at a maximum pairwise genetic distance of 1.5%.

**HIV-1 variant**	**Transmission pairs**	**Pairs per cohort**		**Linked pairs by gender**		
		FCs	GP	M-F	F-F	M-M
Subtype A1	7	4	3	4	1	2
Subtype D	4	4	_	3	1	_
A1/D recombinants	2	2	_	2	_	_
**Total**	**13**	**10**	**3**	**9**	**2**	**2**

Table S3. TMRCA, time depth and genetic distance (cut-off=4.5%) of transmission clusters/pairs

*Cluster/pairs	MRCA (year)	Time depth (years)	GD (%)
**1**	2014.5	1	1.11
**2**	2007.57	1.7	0.08
**3**	2015.06	0.4	0.08
**4**	2006.69	2.7	0.16
**5**	2015.09	0.3	0.08
**6**	2011.56	3.9	1.11
**7**	2006.54	8.4	1.35
**8**	2014.77	0.7	0.16
**9**	2015.06	0.4	0.31
**10**	2015.06	0.4	0.08
**11**	2011.25	3.8	0.64
**12**	2004.94	10	1.59
**13**	2010.14	5.4	1.83
**14**	2011.36	3.6	3.5
**15**	2005.7	9.86	2.78
**16**	1998.96	16.5	3.34
**17**	2014.26	1.1	1.75
**18**	2003.14	12.4	4.3
**19**	1997.85	17.6	4.06
**20**	2002.1	12.9	2.31
**21**	2002.99	12.5	3.42
**22**	1998.31	16.6	3.18
**23**	2011.07	4	4.46
**24**	2009.28	6.2	3.66
**25**	2008.99	6.5	3.02
**26**	2005.7	9.9	2.78
**27**	2003.35	11.6	2.63
**28**	2001.87	13.7	3.98
**29**	2002.22	12.7	3.34
**30**	1998.27	16.6	3.66
**31**	1997.6	17.8	3.66

All nodes were supported at a posterior probability of 1

∗ Pairs 1-11 represented linked individuals at a genetic distance threshold of 1.5%

Table S4: Support for other significant viral migration between locations with BF>3

Viral migration between FCs and GP	Subtype A1	Subtype D
Rakai-FC1	31	19
Kampala-FC2	13	4
FC1-FC3	5	
FC1-FC2	5	
Mpigi-FC1	5	
Mpigi-Wakiso	5	4
Wakiso-FC1	4	
Wakiso-Mpigi	4	
Mpigi-FC3		4
Wakiso-FC3		4
Wakiso-FC2		4
Rakai-FC1		3
Kampala-Wakiso		3
Mpigi-FC2		3

Table S5. Significant viral migration with BF>3 in second phylogeographic analysis

Viral migration between FCs and GP	Subtype A1	Subtype D
Mpigi-FC3	178	51
Mpigi-FC2	177	246
Wakiso-FC3	110	50
Wakiso-FC1	94	98
Mpigi-FC1	52	61

Table S6. HIV-1 Sequences by Location and Subtype Used in the Discrete Traits Analysis

	FCs and GP sites	Subtype A1	Subtype D	Total
1	FC1	33	23	56
2	FC2	29	21	50
3	FC3	28	21	49
4	Mpigi	34	30	64
5	Wakiso	33	30	63
6	Kampala	34	41	75
7	Rakai	17	11	28
	**Total**	**208**	**177**	**385**
